# Environmental, social, and governance information disclosure and stock price crash risk: Evidence from Chinese listed companies

**DOI:** 10.3389/fpsyg.2022.977369

**Published:** 2022-09-20

**Authors:** Nengrui Xu, Jing Liu, Huan Dou

**Affiliations:** ^1^School of Management, Hainan University, Haikou, Hainan, China; ^2^School of Management, Jinan University, Guangzhou, Guangdong, China

**Keywords:** ESG information disclosure, reputational capital, stock price crash risk, information asymmetry theory, stakeholder theory

## Abstract

According to information asymmetry theory and stakeholder theory, this article explores the impact and mechanism of environmental, social, and governance (ESG) information disclosure on the company’s future stock price crash risk based on the A-share listed companies from 2010 to 2019. We find that ESG information disclosure significantly reduces the company’s future stock price crash risk. This conclusion remains robust after a series of robustness tests, such as PSM-DID. The heterogeneity analysis shows that the negative relationship between ESG disclosure and stock price crash risk is more significant in state-owned enterprises, companies with higher agency costs, and when companies in the bull market. The mechanism is that companies choose to disclose ESG information to alleviate information asymmetry problems and enhance corporate reputation capital, thus reducing the future stock price crash risk. This article shows that strengthening ESG construction will help improve the efficiency of China’s resource allocation and promote the capital market development.

## Introduction

In 2004, the United Nations Global Compact first proposed the concept of environmental, social, and governance (ESG). In 2006, the United Nations Principles for Responsible Investment (UN PRI) incorporated environmental, social, and corporate governance into the investor evaluation system, aiming to help investors understand the impact of ESG information on value investing. ESG evaluation mainly measures the economic value, social value, and future sustainable development capability of enterprises, which becomes a new standard for high-quality development.

In recent years, the number of listed companies that disclose ESG information in China has increased. According to SynTao Green Finance’s ESG Rating Report in 2020, the number of ESG reports^1^ disclosed by Chinese A-share listed companies has increased from 471 in 2010 to 1,092 in 2020. The company discloses ESG information to the outside, such as the company’s environmental protection, social responsibility, and corporate governance status. The information not only helps stakeholders form a more comprehensive view and optimize the information environment of the company but also is an integral part of a green and low-carbon circular economy system under the background of China’s “double carbon” goal.

However, since it is voluntary to disclose ESG information in China until now, and there is no standard information disclosure regulation, the management is given much freedom to choose the ESG information disclosure strategy. Based on the information obfuscation hypothesis, the managers who have the motivation to benefit themselves are willing to use ESG information disclosure as a self-interested tool to conceal negative news about the company, such as poor financial performance and unethical behavior (Kim et al., 2014; Liu et al., 2022). Therefore, whether corporate ESG information disclosure can genuinely improve the information quality of the capital market and optimize resource allocation needs further discussion.

High-quality information disclosure is an essential foundation for the healthy operation of the capital market and the improvement of market pricing efficiency. In recent years, it is common for the stock prices of individual stocks in China’s stock market to plummet. For example, in 2015, more than 1,000 stocks fell by the limit, and in 2019, Kangmei Pharmaceutical and Kangdexin committed financial fraud. It has had a tremendous negative impact on the healthy development of China’s stock market and investors’ wealth. Based on the bad news hiding the hypothesis of Jin and Myers (2006), insiders hide bad news for a long time based on self-interest, which increases the degree of information asymmetry between inside and outside of the company. When the bad news can no longer be concealed and released to the market, it will cause a sharp drop in its stock price or even collapse.

At present, scholars have found that financial reporting opacity (Hutton et al., 2009), primary shareholder control rights-cash flow rights separation (Hong et al., 2017), exploratory innovation strategy (Jia, 2018), and powerful CEOs (Al Mamun et al., 2020) have a positive impact on stock price crash risk, while female CFOs (Li and Zeng, 2019), internet searching (Xu et al., 2021), green Commitment (Liu et al., 2022), and bank deregulation (Dang et al., 2022) negatively affect stock price crash risk. Based on the strategic development need of China, this article explores whether and how to optimize the efficiency of resource allocation from the perspective of the stock price collapse, which is of great practical significance.

Using the data of China’s A-share listed companies from 2010 to 2019, we examine the impact of corporate ESG information disclosure on stock price crash risk. We find that the more complete the company discloses ESG information, the more significant it will reduce the risk of future stock price crashes. Further analysis shows that the negative relationship between ESG disclosure and stock price crash risk is more significant in state-owned enterprises, companies with higher agency costs, and when companies in the bull market. Disclosure of ESG information by companies can alleviate information asymmetry and enhance corporate reputation capital, thus reducing the future stock price crash risk.

The research contributes to the following two aspects. First, compared to foreign countries, China has limited empirical attention to ESG, and little literature examines the relationship between corporate ESG information disclosure and stock price crash risk. The existing literature mostly studies the impact on stock price crash risk from the perspectives of company information quality, management motivation, and characteristics, such as information transparency (Hutton et al., 2009), accounting conservatism (Kim and Zhang, 2016), executive gender (Li and Zeng, 2019), etc. We measure ESG information disclosure from the two dimensions of whether companies disclose ESG information and the degree of its disclosure, and explore its impact on stock price crash risk. It complements the literature on the influential factor of the stock price crash risk.

Second, this article finds that ESG information disclosed by companies can alleviate information asymmetry to a certain extent and improve reputational capital. The existing literature generally believes that information asymmetry (Jin and Myers, 2006), agency problems (Li and Zeng, 2019), and irrational behavior of institutional investors (An and Zhang, 2013; Callen and Fang, 2013; Xu et al., 2013), etc., are the important mechanisms that lead to the crash of the stock price of listed companies. We incorporate information transparency and corporate reputation capital into the same research framework, systematically analyze the transmission mechanism of ESG information disclosure to stock price crash risk from a theoretical level, and reveal the inherent logical relationship between ESG information disclosure and stock price crash risk, which will enrich existing research.

## Literature review and hypothesis development

### Environmental, social, and governance-related research

At present, scholars have been actively exploring the field of ESG. Some scholars believe that the ESG practice of enterprises violates the principle of profit maximization, and the company may miss investment opportunities, resulting in inefficient investment portfolios and destroying enterprise value (Fabozzi et al., 2008; Revelli and Viviani, 2015). However, most scholars have found that ESG practices can improve corporate competitive advantage, bring returns to shareholders (Porter and Kramer, 2006), and improve the performance of enterprises (Friede et al., 2015; Mervelskemper and Streit, 2017; Yu et al., 2018).

In addition, scholars have also paid more attention to the issue of how ESG information affects the allocation of market resources, which can be summarized into the following three aspects: first, improve the information content in the market and alleviate information asymmetry. Yuan et al. (2022) studied the relationship between ESG information disclosure and corporate irregularities from the perspective of non-financial information disclosure and found that ESG disclosure can alleviate information asymmetry, improve information transparency, and restrain corporate financial irregularities. Ellili (2022) found that companies’ disclosure of ESG information can not only improve corporate transparency but also help companies make rational investment decisions and improve investment efficiency.

Second, attract the attention of investors and obtain excess investment returns. Cao et al. (2020) found that ESG information disclosed by companies often attracts the attention of social responsibility agencies, which will change investors’ trading behavior, and then have an impact on investors’ stock return patterns and market pricing of information. Pastor et al. (2021) analyzed a two-factor model driven by green factors and found that a portfolio that considered ESG factors could achieve higher stock returns, and green stocks performed better.

Third, gain reputational advantages, reduce default risk, and improve long-term competitiveness. Jahmane and Brahim (2020) and Lemma et al. (2021) found that the “green” behavior of enterprises can send positive signals, form the reputation capital of enterprises, ease the financing constraints of enterprises, and then obtain economic returns. Some scholars have also found that even if a company’s “green” behavior does not yield financial returns immediately (Wan et al., 2021), the accumulated reputational capital can significantly improve the long-term competitiveness of the company and improve the stability of stock prices (Dhaliwal et al., 2011; Liu et al., 2022).

### Hypothesis development

As a kind of non-financial information, ESG reports can enrich the understanding of external investors about the value-relevant information on corporate environmental, social, and corporate governance. This enlightenment can mitigate the degree of information asymmetry, and improve corporate reputation and investor risk tolerance, thus reducing the probability of the company’s future stock price crash.

On the one hand, ESG information disclosure is a useful way to provide the information that external investors’ need, which relieves the information asymmetry in China’s capital market. First, the ESG disclosure integrates the individual information that is beneficial to the company into the stock price, attracts more investor attention (Liu et al., 2022; Tao et al., 2022), and reduces the heterogeneity of investors’ expectations for the company’s stock in future caused by the lack of information (Yuan et al., 2022). Second, companies with better ESG performance are more willing to disclose ESG information (Jagannathan et al., 2017), showing to the outside that the company has a high level of environmental protection responsibility, cultural conservation, and corporate governance. Due to the widespread information discrepancy between inside management and outside investors, company managers have an incentive to use such advantages to benefit themselves. When a company voluntarily discloses ESG information to the outside, the motivation to conceal information is scarce. Therefore, companies’ disclosure of ESG information will help alleviate the agency problem caused by insider control (Liu et al., 2022), improve the transparency of information disclosure, and reduce the information risk faced by investors (Eccles et al., 2014). Third, better communication between the company and investors helps investors obtain a clearer company image alleviating the degree of information asymmetry between inside and outside the company, thereby reducing the risk of future stock price crashes. Further, the company’s ESG performance is relatively good, and it will force companies to improve the level and transparency of ESG information disclosure, which will play a more substantial role in suppressing the collapse of stock prices.

On the other hand, ESG information disclosure is a signal for outsiders that the company cares about the interests of stakeholders, such as employees and customers, which may improve employee satisfaction (Lyon and Montgomery, 2015; El Akremi et al., 2018) and attract more high-quality employees and clients, giving organizations a competitive advantage. First, environmental problems such as global warming caused by excessive carbon emissions endanger the normal life and economic activities of about 1 billion people (Tian et al., 2022). Therefore, when companies disclose ESG information in line with the concept of green and low-carbon development to stakeholders, they not only demonstrate their commitment to environmental and social responsibilities to stakeholders but also send a non-self-interested signal to stakeholders. It helps corporates form an excellent citizenship image. The accumulation of this long-term positive image can improve the enterprise’s reputation (Cho et al., 2012) and form a critical intangible asset in the information asymmetry environment. When negative news flows out of the company, this reputational capital may increase investors’ tolerance for bad news, which will have a specific buffering effect on stock price fluctuations caused by negative news (Cao et al., 2020), like a protection mechanism, to prevent the collapse of the company’s stock price in future. In addition, unlike social responsibility reports, companies that disclose ESG information pursue social benefits and pay attention to their corporate governance level, that is, considering both corporate value and social value. To a certain extent, this can avoid the decline of corporate productivity and the impairment of corporate value due to over-emphasis on social benefits and truly satisfy the interests of multiple stakeholders, including shareholders. ESG information disclosure shows a better governance level and alleviates the information asymmetry problem, which helps attract multiple stakeholders. Under such circumstances, the stock price can reflect the actual production and operation of the company timely, thereby restraining the collapse caused by the excessive deviation between the stock price and the company’s intrinsic value. We propose the following hypothesis:

**Hypothesis 1:** Environmental, social, and governance (ESG) information disclosure can help reduce the company’s future stock price crash risk.

## Research design

### Data and sample selection

Our initial sample consists of all Chinese A-share listed firms in the period 2010–2019. The sample collection processes are as follows: (1) delete firms operating in the financial industry. (2) Delete firms that are ST (special treat) or PT (particular transfer) in the current year. (3) Delete firms with fewer than 30 trading weeks in a year (as per the requirements for calculating the stock price crash risk). (4) Delete firms with missing financial data. (5) All continuous variables are winsorized at the 1 and 99% levels. This process yields a sample comprising 19,056 firm-year observations. The company’s ESG information disclosure score comes from the Bloomberg database, and other data are from the CSMAR database. We use Stata 15.1 to process data.

### Variables definition

#### Stock price crash risk

We follow the literature (Kim et al., 2011; Kim and Zhang, 2016) by using the negative conditional skewness of returns and the down-to-up volatility of returns to measure the stock price crash risk. The specific calculation process is as follows.

First, model (1) eliminates the impact of market factors on the return on individual stocks. Use the weekly rate of return data for stock i to calculate the weekly rate of return for stock i after excluding market influences.


(1) $$R_{\text{i},\text{t}}=\beta_{0}+\beta_{1}\,R_{\text{m},t-2}+\beta_{2}\,R_{\text{m},t-1}+\beta_{3}\,R_{\text{m},\text{t}}+\beta_{4}\,R_{\text{m},\text{t}+1}+\beta_{5}\,R_{\text{m},\text{t}+2}+\varepsilon_{\text{i},\text{t}}$$

where R_*i,t*_ is the stock return on firm i at week t, R_*m,t*_ is the return on the value-weighted market index to which this firm belongs at week t, and ε_*i,t*_ represents an error term. In this article, the lag and advance of the market rate of return are added to model (1) to adjust the impact of non-synchronous transactions. Residual represents a portion of a stock’s yield that cannot be explained by fluctuations in the market’s yield. Following the existing literature, we define the firm-specific weekly returns for firm j in week w (W_*i,t*_) as the natural logarithm of 1 plus the residual [i.e., W_*i,t*_ = ln(1+ε_*i*,*t*_)].


(2) $$NCSKEW_{i,t}=-\left[n{\left(n-1\right)}^{3/2}\sum W_{i,t}^{3}\right]/[\left(n-1\right)\left(n-2\right){\left(\sum W_{i,t}^{2}\right)}^{3/2}]$$

(3) $$D\,U\,V\,O\,L_{i,t}=\log\left\{\left[\left(n_{u}-1\right)\,\sum_{D\,o\,w\,n}W_{i,t}^{2}\right]\,\,/\,\,\left[\left(n_{d}-1\right)\,\sum_{U\,p}W_{i,t}^{2}\right]\right\}$$

Our first proxy for stock price crash risk is the negative conditional firm-specific skewness of weekly return (*NCSKEW*_*i,t*_). *NCSKEW*_*i,t*_ is calculated by taking the negative of the third moment of firm-specific weekly returns for each sample year and dividing it by the standard deviation of firm-specific weekly returns raised to the third power. A higher value for *NCSKEW*_*i,t*_ indicates a higher crash risk.

Our second proxy for stock price crash risk is the down-to-up volatility (*DUVOL_*i,t*_*), which is the log of the ratio of the standard deviation on the down weeks to the standard deviation on the up weeks. In model (3), *n*_*u*_ is the number of “up” weeks and *n*_*d*_ is the number of “down” weeks. A higher value for *DUVOL*_*i,t*_ also indicates a higher crash risk.

#### Environmental, social, and governance information disclosure

Following Lokuwaduge and De Silva (2022), we use the binary dummy variables of whether the company discloses ESG information (*ESG_if_*t*_*) and ESG information disclosure score (*ESG_score_*t*_*) as the proxy variable for ESG information disclosure.^2^ If Bloomberg gives an ESG score to a listed Chinese company i in year t, *ESG_if_*t*_* is 1; otherwise, it is 0; we measure the ESG information disclosure degree (*ESG_score_*t*_*) by using the ESG score released by Bloomberg. The higher the ESG score, the better the completeness and compliance of ESG information disclosed by the company.

#### Other variables

We follow the extant literature (Xu et al., 2013; Kim and Zhang, 2016; Liu et al., 2022), and choose the following variables as our control variables, such as *OTurnover*_*t*_, *Sigma*_*t*_, *Ret*_*t*_, *Size*_*t*_, and so on. In addition, we control for industry and year-fixed effects. The specific variable definitions are listed in Appendix A.

### Empirical model

In order to test the relationship between ESG information disclosure and stock price crash risk, we construct model (4) for empirical test:


(4) $$C\,r\,a\,s\,h_{i,t+1}=\alpha_{0}+\alpha_{1}\,E\,S\,G_{i,t}+\sum_{k=3}^{m}\gamma_{k}\,C\,o\,n\,t\,r\,o\,l\,s_{t}+I\,n\,d\,u\,s\,t\,r\,y+Y\,e\,a\,r+\varepsilon_{i,t}$$

where *Crash*_*i,t*+1_ represents the stock price crash risk of individual stock i in year t+1, respectively, using the negative yield skewness coefficient (*NCSKEW*_*i,t*+1_) of stock i in year t+1 and the yield fluctuations in rates of stock (*DUVOL*_*i,t*+1_) as the proxy variable; *ESG*_*i,t*_ represents the company’s ESG information disclosure, which is measured by whether the company discloses ESG information (*ESG_if_*t*_*) and the degree of ESG information disclosure (*ESG_score_*t*_*); *Controls* represent the control variables in Appendix A, *Industry* and *Year* are dummy variables for industry and year, respectively.

## Empirical results

### Descriptive statistics

Table 1 reports the results of descriptive statistics. The means of *NCSKEW*_*t*+1_ and *DUVOL*_*t*+1_ are −0.309 and −0.202, respectively. The standard deviations are 0.729 and 0.482, respectively. It suggests differences in the degree of stock price crash risk faced by different companies. The mean value of *ESG_if_*t*_* is 0.425, the minimum value is 0, and the median value is 0, indicating that more than half of the sample companies have not disclosed ESG information. The standard deviation of *ESG_if_*t*_* is 0.494, indicating that there are differences in whether the company chooses to disclose ESG information. The mean value of *ESG_score_*t*_* is 0.087, the minimum value is 0, the maximum value is 0.397, and the standard deviation is 0.109, among the companies that disclose ESG information, there are also differences in the degree of disclosure of ESG information. The distributions of the remaining variables are all within a reasonable range.

**TABLE 1:** Summary statistics.

Variable	*N*	Mean	STD	Min	25%	50%	75%	Max
*NCSKEW*_*t*+1_	19056	–0.309	0.729	–2.472	–0.710	–0.266	0.125	1.747
*DUVOL*_*t*+1_	19056	–0.202	0.482	–1.385	–0.522	–0.200	0.117	1.077
*ESG_if_*t*_*	19056	0.425	0.494	0	0	0	1	1
*ESG_score_*t*_*	19056	0.087	0.109	0	0	0	0.190	0.397
*OTurnover*_*t*_	19056	–0.127	0.442	–1.962	–0.264	–0.054	0.085	0.898
*Sigma*_*t*_	19056	0.061	0.023	0.025	0.045	0.055	0.070	0.144
*Ret*_*t*_	19056	0.002	0.009	–0.016	–0.004	0.001	0.007	0.031
*Size*_*t*_	19056	22.356	1.29	19.889	21.447	22.189	23.102	26.245
*BM*_*t*_	19056	0.642	0.246	0.122	0.454	0.649	0.832	1.151
*NCSKEW*_*t*_	19056	–0.271	0.719	–2.396	–0.665	–0.240	0.154	1.747
*Lev*_*t*_	19056	0.46	0.206	0.063	0.300	0.458	0.616	0.918
*ROA*_*t*_	19056	0.04	0.059	–0.22	0.013	0.036	0.067	0.211
*ABACC*_*t*_	19056	0.062	0.064	0	0.019	0.043	0.083	0.35
*Growth*_*t*_	19056	0.172	0.425	–0.563	–0.017	0.104	0.255	2.822
*Age*_*t*_	19056	2.139	0.887	0	1.609	2.398	2.833	3.258
*Indep*_*t*_	19056	0.373	0.053	0.333	0.333	0.333	0.429	0.571
*Board*_*t*_	19056	8.787	1.732	5	8	9	9	15
*Dual*_*t*_	19056	0.227	0.419	0	0	0	0	1
*State*_*t*_	19056	0.448	0.497	0	0	0	1	1

### Regression analysis

Table 2 shows the results of testing the relationship between ESG information disclosure and stock price crash risk using model (4). Columns (1) and (2) are the regression results of whether to disclose ESG information (*ESG_if_*t*_*) and stock price crash risk (*NCSKEW*_*t*+1_ and *DUVOL*_*t*+1_). The results show that the regression coefficient of *ESG_if_*t*_* is significantly negative at the 5% level, indicating that companies choosing to disclose ESG information can reduce the future stock price crash risk; Columns (3) and (4) are the regression results of ESG information disclosure score (*ESG_score_*t*_*) and stock price crash risk (*NCSKEW*_*t*+1_ and *DUVOL*_*t*+1_). The coefficients of *ESG_score_*t*_* are −0.154 and −0.103, respectively, and are significant at the 5% level, indicating that the better the company’s disclosure of ESG information is, the more significant it will reduce the risk of future stock price crashes. The results demonstrate our hypothesis. That is, ESG information disclosure is negatively related to the company’s future stock price crash risk. Economically, for each standard deviation increase in the ESG information disclosure score of a company, the company’s future stock price crash risk *NCSKEW*_*t*+1_ (*DUVOL*_*t*+1_) correspondingly decreases by 0.017 (0.011), which is equivalent to 5.5% (5.4%) of the mean. From the perspective of control variables, the sign and significance of *OTurnover*_*t*_, *Ret*_*t*_, *NCSKEW*_*t*_, and *BM*_*t*_ are consistent with previous studies (Kim et al., 2011; Callen and Fang, 2013; Liu et al., 2022).

**TABLE 2:** Baseline regression results.

Variable	(1)	(2)	(3)	(4)

	*NCSKEW*_*t*+1_	*DUVOL*_*t*+1_	*NCSKEW*_*t*+1_	*DUVOL*_*t*+1_
*ESG_if_*t*_*	−0.028**	−0.019**		
	(−2.143)	(−2.269)		
*ESG_score_*t*_*			−0.154**	−0.103**
			(−2.525)	(−2.540)
*OTurnover*_*t*_	−0.006	−0.003	−0.006	−0.003
	(−0.376)	(−0.319)	(−0.377)	(−0.322)
*Sigma*_*t*_	−0.580	−0.589**	−0.597	−0.600**
	(−1.520)	(−2.322)	(−1.564)	(−2.362)
*Ret*_*t*_	10.853***	7.062***	10.877***	7.079***
	(11.271)	(10.976)	(11.301)	(11.008)
*Size*_*t*_	0.026***	0.007	0.029***	0.008
	(3.536)	(1.396)	(3.737)	(1.621)
*BM*_*t*_	−0.325***	−0.192***	−0.328***	−0.193***
	(−9.367)	(−8.323)	(−9.458)	(−8.382)
*NCSKEW*_*t*_	0.073***	0.046***	0.073***	0.046***
	(9.466)	(8.989)	(9.465)	(8.989)
*Lev*_*t*_	0.034	0.027	0.031	0.025
	(0.955)	(1.160)	(0.873)	(1.086)
*ROA*_*t*_	−0.248**	−0.248***	−0.254**	−0.252***
	(−2.195)	(−3.307)	(−2.243)	(−3.359)
*ABACC*_*t*_	0.366***	0.236***	0.364***	0.234***
	(4.251)	(4.168)	(4.225)	(4.142)
*Growth*_*t*_	−0.009	−0.006	−0.010	−0.006
	(−0.674)	(−0.692)	(−0.717)	(−0.733)
*Age*_*t*_	−0.065***	−0.048***	−0.065***	−0.048***
	(−8.110)	(−9.264)	(−8.121)	(−9.285)
*Indep*_*t*_	−0.082	−0.051	−0.079	−0.049
	(−0.741)	(−0.703)	(−0.713)	(−0.677)
*Board*_*t*_	−0.006	−0.004*	−0.006	−0.004*
	(−1.616)	(−1.883)	(−1.606)	(−1.877)
*Dual*_*t*_	0.008	0.003	0.008	0.003
	(0.621)	(0.375)	(0.600)	(0.356)
*State*_*t*_	−0.044***	−0.023***	−0.043***	−0.022***
	(−3.511)	(−2.771)	(−3.434)	(−2.694)
*Constant*	−0.341**	−0.007	−0.393**	−0.037
	(−2.177)	(−0.064)	(−2.425)	(−0.338)
*Year*	Yes	Yes	Yes	Yes
*Industry*	Yes	Yes	Yes	Yes
*N*	19056	19056	19056	19056
*Adj-R^2^*	0.065	0.069	0.065	0.069

***, **, and * are significant at the level of 0.01, 0.05, and 0.1, respectively.

## Robustness check

In this section, we will conduct a battery of robustness checks on the empirical results, including PSM-DID, instrumental variable method, and alternative measures of dependent variables.

### Propensity score matching-DID

To mitigate potential endogeneity problems caused by sample selection bias, we adopt the propensity score matching (PSM) method for robustness check. The dependent variables in the benchmark regression in this article are lagged by one period, which can alleviate the endogenous problem caused by reverse causality to a certain extent. This article constructs a time-varying DID model (5) to control possible endogenous problems.


(5) $$C\,r\,a\,s\,h_{i,t+1}=\alpha_{0}+\alpha_{1}\,T\,r\,e\,a\,t_{i,t}+\sum_{k=3}^{m}\gamma_{k}\,C\,o\,n\,t\,r\,o\,l\,s_{t}+I\,n\,d\,u\,s\,t\,r\,y+Y\,e\,a\,r+\varepsilon_{i,t}$$

where *Crash-Risk_*i,t*_*_+1_ represents two measures of stock price crash risk (*NCSKEW*_*t*+1_ and *DUVOL*_*t*+1_), *Treat*_*i,t*_ is a binary dummy variable that changes with individuals and time, if company i discloses ESG information in year t, then treat *Treat*_*i,t*_ as 1 for the current year and subsequent years; otherwise, it is assigned as 0. Since different companies have significant differences in company characteristic variables such as size and profitability, this article draws on the research of Rosenbaum and Rubin (1983) to use propensity score matching (PSM) to perform nearest neighbor 1:1 caliper matching to obtain the treatment group and the control group. Then we use model (5) to regress.

Table 3 displays the results. The regression coefficients of *Treat*_*t*_ are all negative and significant at the 10% level at least. It shows that after controlling the possible endogeneity problems, the negative relationship between ESG information disclosure and the future stock price crash risk is still significant, indicating the robustness of the baseline regression results in this article.

**TABLE 3:** Propensity score matching+DID method.

Variable	PSM+DID	
	(1)	(2)

	*NCSKEW*_*t*+1_	*DUVOL*_*t*+1_
*Treat*_*t*_	−0.032*	−0.021**
	(−1.892)	(−1.969)
*Controls*	Yes	Yes
*Constant*	−0.886**	−0.557**
	(−2.300)	(−2.045)
*Year*	Yes	Yes
*Industry*	Yes	Yes
*N*	19023	19023
*Adj-R^2^*	0.069	0.072

***, **, and * are significant at the level of 0.01, 0.05, and 0.1, respectively.

### Instrumental variable method

In order to alleviate the interference of endogenous problems such as measurement error and sample self-selection in the conclusions of this article, we adopt the ratio of the number of ESG information disclosures (IV1) that belong to the same year-industry to the total number of companies and the ratio of the number of ESG information disclosed belong to the same year-province to the total number of companies (IV2) as an instrumental variable for whether to disclose ESG information (*ESG_if_*t*_*). In addition, we use the mean value of ESG information disclosure scores (IV3) belonging to the same year-industry and the mean value of ESG information disclosure score belonging to the same year-province (IV4) as the instrumental variables of ESG information disclosure degree (*ESG_score_*t*_*).

Table 4 presents the regression results of the instrumental variable method. From the regression results of the first stage, the coefficients of the instrumental variables are all significantly positive at the level of 1%, indicating that the instrumental variables can well-explain ESG information disclosure variables (*ESG_if_*t*_* and *ESG_score_*t*_*); the second-stage regression results show that the regression coefficients between *ESG_if_*t*_* and stock price crash risk (*NCSKEW*_*t*+1_ and *DUVOL*_*t*+1_) are significantly negative at the 5% level. The regression coefficient of *ESG_score_*t*_* and stock price crash risk (*NCSKEW*_*t*+1_ and *DUVOL*_*t*+1_) are significantly negative at the 1% level. Moreover, the instrumental variables have passed the weak instrumental variables and over-identification tests. It shows that after alleviating the possible endogenous, the benchmark regression results in this article remain robust.

**TABLE 4:** Instrumental variables method.

Variable	First stage		Second stage			
	(1)	(2)	(3)	(4)	(5)	(6)

	*ESG_if_*t*_*	*ESG_score_*t*_*	*NCSKEW*_*t*+1_	*DUVOL*_*t*+1_	*NCSKEW*_*t*+1_	*DUVOL*_*t*+1_
*ESG_if_*t*_*			−0.303***	−0.134**		
			(−3.648)	(−2.452)		
*ESG_score_*t*_*					−1.317***	−0.622***
					(−4.089)	(−2.948)
*IV*1	0.488***					
	(5.250)					
*IV*2	0.569***					
	(20.349)					
*IV*3		0.323***				
		(13.389)				
*IV*4		0.533***				
		(21.820)				
*Controls*	Yes	Yes	Yes	Yes	Yes	Yes
*Constant*	−5.369***	−1.223***	−1.779***	−0.603**	−1.870***	−0.696**
	(−69.153)	(−73.935)	(−3.922)	(−2.020)	(−4.335)	(−2.453)
*Year*	Yes	Yes	Yes	Yes	Yes	Yes
*Industry*	Yes	Yes	Yes	Yes	Yes	Yes
*N*	19056	19056	19056	19056	19056	19056
*Adj-R* ^2^	0.372	0.440	0.042	0.060	0.047	0.061
*F statistics*	−	−	241.727	241.727	375.200	375.200
*Hansen J*	−	−	0.420 (*p* = 0.5171)	1.595 (*p* = 0.2066)	0.083 (*p* = 0.7738)	0.013 (*p* = 0.9107)

***, **, and * are significant at the level of 0.01, 0.05, and 0.1, respectively.

### Alternative measures

Following the studies of Hutton et al. (2009) and Dang et al. (2022), we change the criteria for judging stock price slumps and set the threshold to 1%. Specifically, we compare the company’s weekly idiosyncratic return with the average annual idiosyncratic return minus 3.09 standard deviations. If the former is lower than the latter, *CRASH* is assigned a value of 1; otherwise, it is 0. Since *CRASH* is a binary dummy variable, we use the logit model to regress. The regression results are shown in Table 5. The results found that the regression coefficients of ESG information disclosure are all significantly negative at the 1% level. The benchmark results remain robust.

**TABLE 5:** Sensitivity tests for dependent variables.

Variable	(1)	(2)

	*CRASH*_*t*+1_	*CRASH*_*t*+1_
*ESG_if_*t*_*	−0.162***	
	(−2.777)	
*ESG_score_*t*_*		−1.125***
		(−4.053)
*Controls*	Yes	Yes
*Constant*	−0.142	−0.689
	(−0.192)	(−0.906)
*Year*	Yes	Yes
*Industry*	Yes	Yes
*N*	19056	19056
*Pseudo R* ^2^	0.021	0.022

***, **, and * are significant at the level of 0.01, 0.05, and 0.1, respectively.

## Heterogeneity analysis

### Capital market condition

Following Kao et al. (1998), if the average monthly market return of stock within 12°months of the current year is greater than 0, the market condition is a bull market. Otherwise, it is a bear market. We determine the sample data years 2012–2015, 2017, and 2019 as bull markets and the remaining years as bear markets. Table 6 shows the results of regression according to differences in market conditions. The results show that the regression coefficient of ESG information disclosure is significantly negative in the bull market, while the coefficient is not significant in the bear market. It shows that ESG information disclosure in a bull market can further alleviate information asymmetry, restrain investors’ irrational investment decisions, and reduce the irrational bubbles in company stock prices, thereby restraining future stock price crashes.

**TABLE 6:** Capital market condition.

Variable	Bull market				Bear market			
	(1)	(2)	(3)	(4)	(5)	(6)	(7)	(8)

	*NCSKEW*_*t*+1_	*DUVOL*_*t*+1_	*NCSKEW*_*t*+1_	*DUVOL*_*t*+1_	*NCSKEW*_*t*+1_	*DUVOL*_*t*+1_	*NCSKEW*_*t*+1_	*DUVOL*_*t*+1_
*ESG_if_*t*_*	−0.030*	−0.019*			−0.017	−0.014		
	(−1.816)	(−1.724)			(−0.814)	(−1.056)		
*ESG_score_*t*_*			−0.173**	−0.100*			−0.089	−0.078
			(−2.194)	(−1.923)			(−0.920)	(−1.212)
*Controls*	Yes	Yes	Yes	Yes	Yes	Yes	Yes	Yes
*Constant*	0.194	0.411***	0.131	0.382***	−1.348***	−0.773***	−1.373***	−0.796***
	(0.988)	(3.093)	(0.646)	(2.786)	(−5.333)	(−4.661)	(−5.247)	(−4.649)
*Year*	Yes	Yes	Yes	Yes	Yes	Yes	Yes	Yes
*Industry*	Yes	Yes	Yes	Yes	Yes	Yes	Yes	Yes
*N*	11724	11724	11724	11724	7332	7332	7332	7332
*Adj-R^2^*	0.068	0.072	0.069	0.072	0.062	0.066	0.063	0.066

***, **, and * are significant at the level of 0.01, 0.05, and 0.1, respectively.

### Property rights nature

The environmental part of ESG information belongs to public goods with unclear property rights. The related problems cannot be entirely solved by market mechanisms but need the government’s intervention (Huang, 2021). Therefore, compared with non-state-owned enterprises, SOEs are more likely to disclose ESG information (Weber, 2014), provide more information about the company’s characteristics to outside the company, and improve the transparency of market information. Meanwhile, compared with non-state-owned enterprises, SOEs have to undertake specific policy tasks and social responsibilities in addition to pursuing economic interests (Leippold et al., 2022).

Table 7 presents the regression results. According to the different nature of property rights, the sample enterprises are divided into state-owned and non-state-owned enterprises, and then model (4) is regressed. The coefficients of *ESG_if_*t*_* and *ESG_score_*t*_* are significantly negative at least at the 10% level in Columns (1) to (4), and the coefficients of *ESG_if_*t*_* and *ESG_score_*t*_* are not significant in Columns (5) to (8), indicating that compared with non-state-owned enterprises, ESG information disclosure has a more significant effect on reducing stock price crash risk in SOEs.

**TABLE 7:** Property rights nature.

Variable	SOE				Non-SOE			
	(1)	(2)	(3)	(4)	(5)	(6)	(7)	(8)

	*NCSKEW*_*t*+1_	*DUVOL*_*t*+1_	*NCSKEW*_*t*+1_	*DUVOL*_*t*+1_	*NCSKEW*_*t*+1_	*DUVOL*_*t*+1_	*NCSKEW*_*t*+1_	*DUVOL*_*t*+1_
*ESG_if_*t*_*	−0.038**	−0.030**			−0.018	−0.010		
	(−2.059)	(−2.505)			(−1.015)	(−0.808)		
*ESG_score_*t*_*			−0.161*	−0.121**			−0.145	−0.083
			(−1.879)	(−2.162)			(−1.645)	(−1.391)
*Controls*	Yes	Yes	Yes	Yes	Yes	Yes	Yes	Yes
*Constant*	−0.282	0.052	−0.309	0.041	−0.539**	−0.122	−0.608**	−0.166
	(−1.277)	(0.357)	(−1.334)	(0.267)	(−2.250)	(−0.762)	(−2.510)	(−1.022)
*Year*	Yes	Yes	Yes	Yes	Yes	Yes	Yes	Yes
*Industry*	Yes	Yes	Yes	Yes	Yes	Yes	Yes	Yes
*N*	8542	8542	8542	8542	10514	10514	10514	10514
*Adj-R^2^*	0.062	0.065	0.061	0.065	0.058	0.064	0.058	0.064

***, **, and * are significant at the level of 0.01, 0.05, and 0.1, respectively.

### Agency costs

The existence of the agency problem makes the management generally have the motive to hide negative news, and the information asymmetry between inside and outside the enterprise will aggravate the accumulation of negative information and increase the risk of a stock price crash (Kim et al., 2011; Kim and Zhang, 2016). ESG information incorporate more idiosyncratic information into the stock price, which help alleviate the information asymmetry, thus decrease the agency costs. Therefore, we use the company’s total asset turnover rate to measure agency costs and divide the sample into a high agency cost group and a low agency cost group according to the median to explore the difference in agency costs. The above treatment will help explore differences in agency costs on the relationship between ESG information disclosure and stock price crash risk.

Table 8 shows the regression results. In the higher agency cost group, the coefficients of ESG information disclosure variables (*ESG_if_*t*_* and *ESG_score_*t*_*) are significantly negative at least at the 5% level, and in the lower agency cost group, their coefficients are negative but not significant. It shows that ESG information disclosure has a more significant inhibitory effect on stock price crash risk in companies with more severe agency problems.

**TABLE 8:** Agency costs.

Variable	High agency cost				Low agency cost			
	(1)	(2)	(3)	(4)	(5)	(6)	(7)	(8)

	*NCSKEW*_*t*+1_	*DUVOL*_*t*+1_	*NCSKEW*_*t*+1_	*DUVOL*_*t*+1_	*NCSKEW*_*t*+1_	*DUVOL*_*t*+1_	*NCSKEW*_*t*+1_	*DUVOL*_*t*+1_
*ESG_if_*t*_*	−0.054***	−0.031***			−0.004	−0.009		
	(−2.812)	(−2.531)			(−0.256)	(−0.736)		
*ESG_score_*t*_*			−0.251***	−0.150**			−0.062	−0.057
			(−2.756)	(−2.520)			(−0.749)	(−1.043)
*Controls*	Yes	Yes	Yes	Yes	Yes	Yes	Yes	Yes
*Constant*	−0.710***	−0.207	−0.746***	−0.232	0.172	0.284*	0.115	0.254*
	(−3.041)	(−1.352)	(−3.095)	(−1.471)	(0.809)	(1.945)	(0.521)	(1.690)
*Year*	Yes	Yes	Yes	Yes	Yes	Yes	Yes	Yes
*Industry*	Yes	Yes	Yes	Yes	Yes	Yes	Yes	Yes
*N*	9528	9528	9528	9528	9528	9528	9528	9528
*Adj-R^2^*	0.062	0.069	0.062	0.069	0.071	0.072	0.071	0.072

***, **, and * are significant at the level of 0.01, 0.05, and 0.1, respectively.

## Mechanism analysis

In this section, we try to provide the mechanism about why the ESG information disclosure is negatively related to stock price crash risk.

### Degree of information asymmetry

The disclosure of ESG information by companies may alleviate the information asymmetry between inside and outside the company to a certain extent (Lyon and Montgomery, 2015; Yuan et al., 2022), improve the pricing efficiency of the capital market, and then reduce the company’s future stock price crash risk. Therefore, we draw on the mediation effect test procedure of Wen et al. (2004) to examine whether information asymmetry plays a mediating role in the relationship between ESG information disclosure and stock price crash risk. Specifically, following Kaeck et al. (2022), we adopt relative bid-ask spread (*ESP*) as proxy variables for the degree of information asymmetry. The specific calculation is shown in formulas (6).


(6) $$E\,S\,P_{i,t}=\left(\sum_{d=1}^{n}\frac{\left|Price_{i,d}-mid\left(Ask_{i,d},Bid_{i,d}\right)|\times 2\right)}{m\,i\,d\,\left(A\,s\,k_{i,d},B\,i\,d_{i,d}\right)}\right)\,/\,\text{n}$$

where *Ask*_*i,d*_ represents the selling price on the d-th trading day, calculated by the time interval weighted by the time interval between two adjacent transaction records. *Bid*_*i,d*_ represents the bid price on the d-th trading day, weighted by the time interval between two adjacent transaction records. mid(*Ask*_*i, d*_, *Bid*_*i, d*_) represents the midpoint price. n represents the actual number of trading days in year t. The larger the relative effective spread, the higher the degree of information asymmetry.

The regression results are shown in Table 9. The results of the first two columns show that the regression coefficients of ESG information disclosed (*ESG_if_*t*_* and *ESG_score_*t*_*) and *ESP*_*t*+1_ are significantly negative at the 1% level. It shows that the company’s disclosure of ESG information alleviates the degree of information asymmetry. Columns (3) to (6) are the regression results of ESG information disclosed (independent variable) and *ESP*_*t*+1_ (mediating variable) with stock price crash risk (dependent variable). It shows that the company’s disclosure of ESG information alleviates the degree of information asymmetry in the market, thereby reducing the company’s future stock price crash risk.

**TABLE 9:** Mechanism analysis: Information asymmetry.

Variable	Information asymmetry					
	(1)	(2)	(3)	(4)	(5)	(6)

	*ESP*_*t*+1_	*ESP*_*t*+1_	*NCSKEW*_*t*+1_	*DUVOL*_*t*+1_	*NCSKEW*_*t*+1_	*DUVOL*_*t*+1_
*ESG_if_*t*_*	−0.004***		−0.029**	−0.020**		
	(−3.554)		(−2.234)	(−2.360)		
*ESG_score_*t*_*		−0.021***			−0.160***	−0.106***
		(−4.155)			(−2.617)	(−2.632)
*ESP*_*t*+1_			0.682***	0.450***	0.682***	0.450***
			(6.371)	(6.494)	(6.376)	(6.499)
*Controls*	Yes	Yes	Yes	Yes	Yes	Yes
*Constant*	0.418***	0.411***	−0.013	0.210*	−0.066	0.179
	(30.028)	(29.047)	(−0.077)	(1.892)	(−0.383)	(1.573)
*Year*	Yes	Yes	Yes	Yes	Yes	Yes
*Industry*	Yes	Yes	Yes	Yes	Yes	Yes
*N*	19056	19056	19056	19056	19056	19056
*Pseudo R* ^2^ */Adj-R* ^2^	0.371	0.372	0.067	0.071	0.067	0.071
*Sobel test*	−	−	0.075	0.074	0.091	0.090

***, **, and * are significant at the level of 0.01, 0.05, and 0.1, respectively.

### Reputation capital

The company’s disclosure of ESG information reflects the company’s commitment to the environment and social responsibility, which can improve the confidence and recognition of stakeholders in the company, help the company to establish a good corporate citizenship image, and form the reputation capital of the company to deal with the company’s future stock price fluctuations. Therefore, we use the mediation effect model to examine whether reputational capital plays a mediating role in the relationship between ESG information disclosure and stock price crash risk. Regarding the measurement of corporate reputation capital, we collect the “Most Admired Chinese Companies” All-Star List and Industry List released by Fortune (Chinese version) magazine in 2010 as the basis for judgment. Specifically, if the company enters any list in year t, the reputation capital *SY*_*t*_ is taken as 1; otherwise, it is 0.

The regression results are shown in Table 10. Column (1) is the regression result of *ESG_if_*t*_* (independent variable) and reputation capital (dependent variable). The regression coefficient of *ESG_if_*t*_* is positive but not significant. It shows that in the early stage of China’s ESG construction, it may be difficult to form reputation capital only based on whether companies disclose ESG information. Column (2) is the regression result of *ESG_score_*t*_* (independent variable) and *SY*_*t*+1_ (dependent variable), indicating that the more ESG information disclosed by the company, the higher the integrity, and the more significant the improvement of corporate reputation capital; Columns (3) and (4) are the regression results of *ESG_score_*t*_* (independent variable) and *SY*_*t*+1_ (mediating variable) with stock price crash risk (dependent variable). The results show that the more ESG information disclosure, the lower the stock price crash risk; the higher the company’s reputational capital, the lower the stock price crash risk. The *p*-values of Sobel’s mediating effect test are 0.033 and 0.041, indicating that the mediating effect of reputation capital exists. Reputation capital plays a partial intermediary role between the degree of ESG information disclosure and stock price crash risk.

**TABLE 10:** Mechanism analysis: Reputation capital.

Variable	Reputation capital			
	(1)	(2)	(3)	(4)

	*SY*_*t*+1_	*SY*_*t*+1_	*NCSKEW*_*t*+1_	*DUVOL*_*t*+1_
*ESG_if_*t*_*	0.266			
	(1.039)			
*ESG_score_*t*_*		2.856***	−0.151**	−0.101**
		(3.516)	(−2.476)	(−2.492)
*SY_*t*+1_*			−0.094***	−0.062**
			(−2.673)	(−2.503)
*Controls*	Yes	Yes	Yes	Yes
*Constant*	−56.029***	−53.232***	−0.487***	−0.098
	(−24.666)	(−22.586)	(−2.903)	(−0.877)
*Year*	Yes	Yes	Yes	Yes
*Industry*	Yes	Yes	Yes	Yes
*N*	16470	16470	19056	19056
*Pseudo R^2^/Adj-R^2^*	0.513	0.516	0.065	0.069
*Sobel test*	−	−	0.033	0.041

***, **, and * are significant at the level of 0.01, 0.05, and 0.1, respectively.

## Conclusion

The “14th Five-Year Plan” clearly puts forward requirements for society to coordinate environmental, social, and governance benefits, improve the ESG construction system, and in-depth ESG practice. As the first step of the ESG construction system, ESG information disclosure is also a critical step. Whether it can promote the high-quality development of the capital market is worth exploring. Therefore, we take China’s Shanghai and Shenzhen A-share listed companies from 2010 to 2019 as the research object and examine the impact and role of corporate ESG information disclosure on stock price crash risk. The results show that: (1) the more complete the company discloses ESG information, the more significant it will reduce the risk of future stock price crash; (2) in the bull market, SOEs and companies with high agency costs, ESG information disclosure has a more significant impact on reducing the risk of stock price crash; (3) the higher the company’s ESG information disclosure, the lower the information asymmetry and the enhancement of corporate’s reputation capital, thereby restraining the company’s stock price crash risk.

The inspiration for this article mainly includes the following two aspects. First, ESG is an essential part of the construction of a green and low-carbon circular economy system under the background of “double carbon” in China, and it is the starting point for implementing the new development concept and promoting high-quality development of the capital market. Therefore, accelerating the construction of ESG is an urgent need to implement China’s green development concept and adapt to the international environment. This article sorts out the micro-action mechanism of ESG information disclosure and provides new empirical evidence for researching the economic consequences of ESG information disclosure in Chinese academic circles. Further, considering the differences in the nature of property rights and improving the level of corporate governance are both important ways to strengthen the ESG construction system. This will lay the foundation for the sustainable development of enterprises.

Second, in China, where the capital market is developing, the stock price crash has always been the focus of scholars. In our study, we have some suggestions. First, the companies should change the view that disclosing ESG information is just a burden but realize the benefits of lowering the future stock price crash risk and promoting the company’s sustainable development. Second, from the perspective of external market participants, the information disclosed by the company should be treated appropriately. At the same time, it is necessary to strengthen the study of ESG evaluation-related knowledge. Investors should try to avoid excessive interpretation or insufficient analysis of the information disclosed by the company, which will reduce the quality of market information and distort the capital market. Finally, regulators should strengthen the construction of ESG information disclosure evaluation system and review system, and improve the formulation of standard ESG reports as soon as possible.

## Data availability statement

The original contributions presented in this study are included in the article/supplementary material, further inquiries can be directed to the corresponding author.

## Author contributions

NX and JL organized the database and performed the statistical analysis. All authors contributed to the conception and design of the manuscript, wrote the first draft of the manuscript, contributed to manuscript revision, read, and approved the submitted version.

## Footnotes

1 The so-called “ESG report” includes the reports issued by the company under the names of “Social Responsibility Report” and “Sustainability Report.”

2 The so-called “ESG report” includes the reports issued by the company under the names of “Social Responsibility Report” and “Sustainability Report.”

## Appendix A

**TABLE A1:** Variable definitions.

Variable type	Variable name	Variable definitions
Dependent	*NCSKEW*_*t*+1_	The negative yield skewness coefficient of the company’s stock in year t+1, see the text and formula (2) for the algorithm
	*DUVOL*_*t*+1_	The yield fluctuations in rates of the company’s stock return in year t+1, see the text and formula (3) for the algorithm
Independent	*ESG_if_*t*_*	The ESG information disclosed by the company in year t is 1, otherwise it is 0
	*ESG_score_*t*_*	Bloomberg’s ESG score for companies in year t/100
Controls	*OTurnover*_*t*_	The company’s average monthly turnover rate in year t - the average monthly turnover rate in year t-1
	*Sigma*_*t*_	The standard deviation of the company’s weekly returns in year t
	*Ret*_*t*_	The company’s average weekly rate of return in year t
	*Size*_*t*_	The natural logarithm of the firm’s total assets in year t
	*BM*_*t*_	Net assets of the company in year t/(stock price at the end of year t * number of tradable shares + net assets per share * number of non-tradable shares)
	*NCSKEW*_*t*_	The firm’s negative yield skewness coefficient in period t
	*Lev*_*t*_	The company’s total liabilities/total assets in year t
	*ROA*_*t*_	The company’s net profit/total assets in year t
	*ABACC*_*t*_	Absolute value of the modified Jones model residuals
	*Growth*_*t*_	The company’s main business revenue growth rate in year t
	*Age*_*t*_	Ln (ESG information disclosure year–listed year)
	*Indep*_*t*_	Number of independent directors/number of directors of the company in year t
	*Board*_*t*_	The total number of directors of the company in year t
	*Dual*_*t*_	If the chairman of the company concurrently serves as the general manager in year t, it will be 1, otherwise 0
	*State*_*t*_	If the company is a state-owned enterprise in year t, it will be 1,otherwise 0
	*Year*	Dummy variables based on year of ESG disclosure
	*Industry*	According to the dummy variables set by the China Securities Regulatory Commission in 2012, the first two codes are used as the classification standard for manufacturing, and the first code is used as the classification standard for non-manufacturing industries.
